# Supplementary material for: Alzheimer’s diseases in America, Europe, and Asian regions: a global genetic variation
Source: PeerJ. 2024 May 13;12:e17339. doi: 10.7717/peerj.17339 (PMC11097964; doi:10.7717/peerj.17339)
Supplement: Supplemental Information 1 — The raw data shows global genetic variation of “Alzheimer’s diseases in America, Europe, and Asian regions. [file peerj-12-17339-s001.pdf]

100 Articles identified by PubMed and Google Scholars databases

1. Adeyemo, A., & Rotimi, C. (2009). Genetic variants associated with complex human diseases show wide variation across multiple populations. *Public health genomics*, *13*(2), 72-79.
2. Almeida, J. F. F., L. R. Dos Santos, M. Trancozo and F. de Paula. 2018. "Updated meta-analysis of BIN1, CR1, MS4A6A, CLU, and ABCA7 variants in Alzheimer's disease." *Journal of Molecular Neuroscience* 64(3): 471-477.
3. An N, Fu Y, Shi J, Guo HN, Yang ZW, Li YC, Li S, Wang Y, Yao ZJ, Hu B; Alzheimer's Disease Neuroimaging Initiative. Synergistic Effects of APOE and CLU May Increase the Risk of Alzheimer's Disease: Acceleration of Atrophy in the Volumes and Shapes of the Hippocampus and Amygdala. *J Alzheimers Dis*. 2021;80(3):1311-1327. doi: 10.3233/JAD-201162. PMID: 33682707.
4. Andrews, S. J., Fulton-Howard, B., & Goate, A. (2019). Protective variants in Alzheimer's disease. *Current genetic medicine reports*, *7*, 1-12.
5. Bao, J., Wang, X. J., & Mao, Z. F. (2016). Associations between genetic variants in 19p13 and 19q13 regions and susceptibility to Alzheimer disease: A meta-analysis. *Medical science monitor: international medical journal of experimental and clinical research*, *22*, 234.
6. Belin, A. C., B. F. Björk, M. Westerlund, D. Galter, O. Sydow, C. Lind, K. Pernold, L. Rosvall, A. Håkansson and B. Winblad. 2007. "Association study of two genetic variants in mitochondrial transcription factor A (TFAM) in Alzheimer's and Parkinson's disease." *Neuroscience letters* 420(3): 257-262.
7. Benitez BA, Jin SC, Guerreiro R, Graham R, Lord J, Harold D, Sims R, Lambert JC, Gibbs JR, Bras J, Sassi C, Harari O, Bertelsen S, Lupton MK, Powell J, Bellenguez C, Brown K, Medway C, Haddick PC, van der Brug MP, Bhangale T, Ortmann W, Behrens T, Mayeux R, Pericak-Vance MA, Farrer LA, Schellenberg GD, Haines JL, Turton J, Braae A, Barber I, Fagan AM, Holtzman DM, Morris JC; 3C Study Group; EADI consortium; Alzheimer's Disease Genetic Consortium (ADGC); Alzheimer's Disease Neuroimaging Initiative (ADNI); GERAD Consortium; Williams J, Kauwe JS, Amouyel P, Morgan K, Singleton A, Hardy J, Goate AM, Cruchaga C. Missense variant in TREML2 protects against Alzheimer's disease. *Neurobiol Aging*. 2014 Jun;35(6):1510.e19-26. doi: 10.1016/j.neurobiolaging.2013.12.010. Epub 2013 Dec 21. PMID: 24439484; PMCID: PMC3961557.
8. Bertram, L. et al. Genome-wide association analysis reveals putative Alzheimer's disease susceptibility loci in addition to *APOE*. *Am. J. Hum. Genet.* **83**, 623–632 (2008).
9. Biffi, A., J. Shulman, J. Jagiella, L. Cortellini, A. Ayres, K. Schwab, D. Brown, S. Silliman, M. Selim and B. Worrall. 2012. "Genetic variation at CR1 increases risk of cerebral amyloid angiopathy." *Neurology* 78(5): 334-341.
10. Bonham LW, Sirkis DW, Fan J, Aparicio RE, Tse M, Ramos EM, Wang Q, Coppola G, Rosen HJ, Miller BL, Yokoyama JS. Identification of a rare coding variant in TREM2 in a

Chinese individual with Alzheimer's disease. *Neurocase*. 2017 Feb;23(1):65-69. doi: 10.1080/13554794.2017.1294182. PMID: 28376694; PMCID: PMC5639900.

11. Bucossi S, Polimanti R, Ventriglia M, Mariani S, Siotto M, Ursini F, Trotta L, Scrascia F, Callea A, Vernieri F, Squitti R. Intronic rs2147363 variant in ATP7B transcription factor-binding site associated with Alzheimer's disease. *J Alzheimers Dis*. 2013;37(2):453-9. doi: 10.3233/JAD-130431. PMID: 23948886.
12. Carrasquillo, M. M., O. Belbin, T. A. Hunter, L. Ma, G. D. Bisceglio, F. Zou, J. E. Crook, V. S. Pankratz, S. B. Sando and J. O. Aasly. 2011. "Replication of EPHA1 and CD33 associations with late-onset Alzheimer's disease: a multi-centre case-control study." *Molecular neurodegeneration* 6(1): 1-9.
13. Chen Q, Liang B, Wang Z, Cheng X, Huang Y, Liu Y, Huang Z. Influence of four polymorphisms in ABCA1 and PTGS2 genes on risk of Alzheimer's disease: a meta-analysis. *Neurol Sci*. 2016 Aug;37(8):1209-20. doi: 10.1007/s10072-016-2579-9. Epub 2016 May 23. PMID: 27215623.
14. Chung, S. J., J.-H. Lee, S. Y. Kim, S. You, M. J. Kim, J.-Y. Lee and J. Koh. 2013. "Association of GWAS top hits with late-onset Alzheimer disease in Korean population." *Alzheimer Disease & Associated Disorders* 27(3): 250-257.
15. Chung, S. J., M.-J. Kim, J. Kim, Y. J. Kim, S. You, J. Koh, S. Y. Kim and J.-H. Lee. 2014. "Exome array study did not identify novel variants in Alzheimer's disease." *Neurobiology of aging* 35(8): 1958. e1913-1958. e1914.
16. Colonna, M., & Wang, Y. (2016). TREM2 variants: new keys to decipher Alzheimer disease pathogenesis. *Nature Reviews Neuroscience*, *17*(4), 201-207.
17. Cukier, H. N., Kunkle, B. W., Vardarajan, B. N., Rolati, S., Hamilton-Nelson, K. L., Kohli, M. A., ... & Alzheimer's Disease Genetics Consortium. (2016). ABCA7 frameshift deletion associated with Alzheimer disease in African Americans. *Neurology Genetics*, *2*(3), e79.
18. Cuyvers E, De Roeck A, Van den Bossche T, Van Cauwenberghe C, Bettens K, Vermeulen S, Mattheijssens M, Peeters K, Engelborghs S, Vandenbulcke M, Vandenberghe R, De Deyn PP, Van Broeckhoven C, Sleegers K. Mutations in ABCA7 in a Belgian cohort of Alzheimer's disease patients: a targeted resequencing study. *Lancet Neurol*. 2015 Aug;14(8):814-822. doi: 10.1016/S1474-4422(15)00133-7. Epub 2015 Jun 30. PMID: 26141617.
19. De Roeck A, Van Broeckhoven C, Sleegers K. The role of ABCA7 in Alzheimer's disease: evidence from genomics, transcriptomics and methylomics. *Acta Neuropathol*. 2019 Aug;138(2):201-220. doi: 10.1007/s00401-019-01994-1. Epub 2019 Mar 22. PMID: 30903345; PMCID: PMC6660495.
20. Deng, Y.-L., L.-H. Liu, Y. Wang, H.-D. Tang, R.-J. Ren, W. Xu, J.-F. Ma, L.-L. Wang, J.-P. Zhuang and G. Wang. 2012. "The prevalence of CD33 and MS4A6A variant in Chinese Han population with Alzheimer's disease." *Human genetics* 131(7): 1245-1249.
21. Edland, S. D., Tobe, V. O., Rieder, M. J., Bowen, J. D., McCormick, W., Teri, L., ... & Kukull, W. A. (2002). Mitochondrial genetic variants and Alzheimer disease: a case-control

study of the T4336C and G5460A variants. *Alzheimer Disease & Associated Disorders*, *16*(1), 1-7.

22. Fehér Á, Giricz Z, Juhász A, Pákáski M, Janka Z, Kálmán J. ABCA1 rs2230805 and rs2230806 common gene variants are associated with Alzheimer's disease. *Neurosci Lett*. 2018 Jan 18;664:79-83. doi: 10.1016/j.neulet.2017.11.027. Epub 2017 Nov 10. PMID: 29133174.
23. Fehér Á, Juhász A, László A, Pákáski M, Kálmán J, Janka Z. Association between the ABCG2 C421A polymorphism and Alzheimer's disease. *Neurosci Lett*. 2013 Aug 29;550:51-4. doi: 10.1016/j.neulet.2013.06.044. Epub 2013 Jul 1. PMID: 23827224.
24. Fehér Á, Juhász A, Pákáski M, Kálmán J, Janka Z. Genetic analysis of the RELN gene: Gender specific association with Alzheimer's disease. *Psychiatry Res*. 2015 Dec 15;230(2):716-8. doi: 10.1016/j.psychres.2015.09.021. Epub 2015 Sep 13. PMID: 26384575.
25. Ferrari R, Moreno JH, Minhajuddin AT, O'Bryant SE, Reisch JS, Barber RC, Momeni P. Implication of common and disease specific variants in CLU, CR1, and PICALM. *Neurobiol Aging*. 2012 Aug;33(8):1846.e7-18. doi: 10.1016/j.neurobiolaging.2012.01.110. Epub 2012 Mar 7. PMID: 22402018.
26. Fonseca MI, Chu S, Pierce AL, Brubaker WD, Hauhart RE, Mastroeni D, Clarke EV, Rogers J, Atkinson JP, Tenner AJ. Analysis of the Putative Role of CR1 in Alzheimer's Disease: Genetic Association, Expression and Function. *PLoS One*. 2016 Feb 25;11(2):e0149792. doi: 10.1371/journal.pone.0149792. PMID: 26914463; PMCID: PMC4767815.
27. Gaj P, Paziewska A, Bik W, Dąbrowska M, Baranowska-Bik A, Styczynska M, Chodakowska-Żebrowska M, Pfeffer-Baczuk A, Barcikowska M, Baranowska B, Ostrowski J. Identification of a late onset Alzheimer's disease candidate risk variant at 9q21.33 in Polish patients. *J Alzheimers Dis*. 2012;32(1):157-68. doi: 10.3233/JAD-2012-120520. PMID: 22785395.
28. Guerreiro, R., Wojtas, A., Bras, J., Carrasquillo, M., Rogaeva, E., Majounie, E., ... & Hardy, J. (2013). TREM2 variants in Alzheimer's disease. *New England Journal of Medicine*, *368*(2), 117-127.
29. Han Z, Qu J, Zhao J, Zou X. Analyzing 74,248 Samples Confirms the Association Between CLU rs11136000 Polymorphism and Alzheimer's Disease in Caucasian But Not Chinese population. *Sci Rep*. 2018 Jul 23;8(1):11062. doi: 10.1038/s41598-018-29450-2. PMID: 30038359; PMCID: PMC6056482.
30. Harold, D., Abraham, R., Hollingworth, P., Sims, R., Gerrish, A., Hamshere, M. L., ... & Williams, J. (2009). Genome-wide association study identifies variants at CLU and PICALM associated with Alzheimer's disease. *Nature genetics*, *41*(10), 1088-1093.
31. Harper JD, Fan KH, Aslam MM, Snitz BE, DeKosky ST, Lopez OL, Feingold E, Kamboh MI. Genome-Wide Association Study of Incident Dementia in a Community-Based Sample of Older Subjects. *J Alzheimers Dis*. 2022;88(2):787-798. doi: 10.3233/JAD-220293. PMID: 35694926; PMCID: PMC9359180.

32. He L, Loika Y, Park Y; Genotype Tissue Expression (GTEx) consortium; Bennett DA, Kellis M, Kulminski AM; Alzheimer's Disease Neuroimaging Initiative. Exome-wide age-of-onset analysis reveals exonic variants in ERN1 and SPPL2C associated with Alzheimer's disease. *Transl Psychiatry*. 2021 Feb 26;11(1):146. doi: 10.1038/s41398-021-01263-4. PMID: 33637690; PMCID: PMC7910483.
33. Hollingworth, P., D. Harold, R. Sims, A. Gerrish, J.-C. Lambert, M. M. Carrasquillo, R. Abraham, M. L. Hamshere, J. S. Pahwa and V. Moskvina. 2011. "Common variants at ABCA7, MS4A6A/MS4A4E, EPHA1, CD33 and CD2AP are associated with Alzheimer's disease." *Nature genetics* 43(5): 429-435.
34. Huang, R., J. Huang, H. Cathcart, S. Smith and S. Poduslo. 2007. "Genetic variants in brain-derived neurotrophic factor associated with Alzheimer's disease." *Journal of medical genetics* 44(2): e66-e66.
35. Huentelman M, De Both M, Jepsen W, Piras IS, Talboom JS, Willeman M, Reiman EM, Hardy J, Myers AJ. Common BACE2 Polymorphisms are Associated with Altered Risk for Alzheimer's Disease and CSF Amyloid Biomarkers in APOE ε4 Non-Carriers. *Sci Rep*. 2019 Jul 3;9(1):9640. doi: 10.1038/s41598-019-45896-4. PMID: 31270419; PMCID: PMC6610620.
36. Javor J, Ďurmanová V, Párnická Z, Minárik G, Králová M, Pečeňák J, Vašečková B, Režnáková V, Šutovský S, Gmitterová K, Hromádka T, Peterajová Ľ, Shawkatová I. Association of CD33 rs3865444:C>A polymorphism with a reduced risk of late-onset Alzheimer's disease in Slovaks is limited to subjects carrying the APOE ε4 allele. *Int J Immunogenet*. 2020 Oct;47(5):397-405. doi: 10.1111/iji.12489. Epub 2020 Apr 24. PMID: 32333488.
37. Jia L, Li F, Wei C, Zhu M, Qu Q, Qin W, Tang Y, Shen L, Wang Y, Shen L, Li H, Peng D, Tan L, Luo B, Guo Q, Tang M, Du Y, Zhang J, Zhang J, Lyu J, Li Y, Zhou A, Wang F, Chu C, Song H, Wu L, Zuo X, Han Y, Liang J, Wang Q, Jin H, Wang W, Lü Y, Li F, Zhou Y, Zhang W, Liao Z, Qiu Q, Li Y, Kong C, Li Y, Jiao H, Lu J, Jia J. Prediction of Alzheimer's disease using multi-variants from a Chinese genome-wide association study. *Brain*. 2021 Apr 12;144(3):924-937. doi: 10.1093/brain/awaa364. PMID: 33188687; PMCID: PMC8041344.
38. Jiang T, Yu JT, Wang YL, Wang HF, Zhang W, Hu N, Tan L, Sun L, Tan MS, Zhu XC, Tan L. The genetic variation of ARRB2 is associated with late-onset Alzheimer's disease in Han Chinese. *Curr Alzheimer Res*. 2014 May;11(4):408-12. doi: 10.2174/1567205011666140317095014. PMID: 24635845.
39. Jin, S. C., Benitez, B. A., Karch, C. M., Cooper, B., Skorupa, T., Carrell, D., ... & Cruchaga, C. (2014). Coding variants in TREM2 increase risk for Alzheimer's disease. *Human molecular genetics*, *23*(21), 5838-5846.
40. Kamboh MI, Minster RL, Demirci FY, Ganguli M, Dekosky ST, Lopez OL, Barmada MM. Association of CLU and PICALM variants with Alzheimer's disease. *Neurobiol Aging*.

2012 Mar;33(3):518-21. doi: 10.1016/j.neurobiolaging.2010.04.015. Epub 2010 Jun 8. PMID: 20570404; PMCID: PMC3010357.

41. Keenan BT, Shulman JM, Chibnik LB, Raj T, Tran D, Sabuncu MR; Alzheimer's Disease Neuroimaging Initiative; Allen AN, Corneveaux JJ, Hardy JA, Huentelman MJ, Lemere CA, Myers AJ, Nicholson-Weller A, Reiman EM, Evans DA, Bennett DA, De Jager PL. A coding variant in CR1 interacts with APOE-ε4 to influence cognitive decline. *Hum Mol Genet*. 2012 May 15;21(10):2377-88. doi: 10.1093/hmg/dds054. Epub 2012 Feb 17. PMID: 22343410; PMCID: PMC3335317.
42. Kettunen P, Larsson S, Holmgren S, Olsson S, Minthon L, Zetterberg H, Blennow K, Nilsson S, Sjölander A. Genetic variants of GSK3B are associated with biomarkers for Alzheimer's disease and cognitive function. *J Alzheimers Dis*. 2015;44(4):1313-22. doi: 10.3233/JAD-142025. PMID: 25420549.
43. Kim YE, Cho H, Kim HJ, Na DL, Seo SW, Ki CS. PSEN1 variants in Korean patients with clinically suspicious early-onset familial Alzheimer's disease. *Sci Rep*. 2020 Feb 26;10(1):3480. doi: 10.1038/s41598-020-59829-z. PMID: 32103039; PMCID: PMC7044324.
44. Kretzschmar GC, Antoniazzi AAH, Oliveira LC, Nisihara RM, Petzl-Erler ML, de Souza RLR, Boldt ABW. First Report of CR1 Polymorphisms and Soluble CR1 Levels Associated with Late Onset Alzheimer's Disease (LOAD) in Latin America. *J Mol Neurosci*. 2020 Sep;70(9):1338-1344. doi: 10.1007/s12031-020-01547-2. Epub 2020 May 9. PMID: 32388800.
45. Kucukkilic E, Brookes K, Barber I, Guetta-Baranes T; ARUK Consortium; Morgan K, Hollox EJ. Complement receptor 1 gene (CR1) intragenic duplication and risk of Alzheimer's disease. *Hum Genet*. 2018 Apr;137(4):305-314. doi: 10.1007/s00439-018-1883-2. Epub 2018 Apr 19. PMID: 29675612; PMCID: PMC5937907.
46. Kuksa PP, Liu CL, Fu W, Qu L, Zhao Y, Katanic Z, Clark K, Kuzma AB, Ho PC, Tzeng KT, Valladares O, Chou SY, Naj AC, Schellenberg GD, Wang LS, Leung YY. Alzheimer's Disease Variant Portal: A Catalog of Genetic Findings for Alzheimer's Disease. *J Alzheimers Dis*. 2022;86(1):461-477. doi: 10.3233/JAD-215055. PMID: 35068457; PMCID: PMC9028687.
47. Lambert, J. C., Heath, S., Even, G., Campion, D., Sleegers, K., Hiltunen, M., ... & Amouyel, P. (2009). Genome-wide association study identifies variants at CLU and CR1 associated with Alzheimer's disease. *Nature genetics*, *41*(10), 1094-1099.
48. Lambert, J.-C., C. A. Ibrahim-Verbaas, D. Harold, A. C. Naj, R. Sims, C. Bellenguez, G. Jun, A. L. DeStefano, J. C. Bis and G. W. Beecham. 2013. "Meta-analysis of 74,046 individuals identifies 11 new susceptibility loci for Alzheimer's disease." *Nature genetics* 45(12): 1452-1458.
49. Lee JH, Barral S, Reitz C. The neuronal sortilin-related receptor gene SORL1 and late-onset Alzheimer's disease. *Curr Neurol Neurosci Rep*. 2008 Sep;8(5):384-91. doi: 10.1007/s11910-008-0060-8. PMID: 18713574; PMCID: PMC2694663.

50. Lee JH, Cheng R, Barral S, Reitz C, Medrano M, Lantigua R, Jiménez-Velazquez IZ, Rogaeva E, St George-Hyslop PH, Mayeux R. Identification of novel loci for Alzheimer disease and replication of CLU, PICALM, and BIN1 in Caribbean Hispanic individuals. *Arch Neurol*. 2011 Mar;68(3):320-8. doi: 10.1001/archneurol.2010.292. Epub 2010 Nov 8. PMID: 21059989; PMCID: PMC3268783.
51. Li K, Dai D, Zhao B, Yao L, Yao S, Wang B, Yang Z. Association between the RAGE G82S polymorphism and Alzheimer's disease. *J Neural Transm (Vienna)*. 2010 Jan;117(1):97-104. doi: 10.1007/s00702-009-0334-6. Epub 2009 Nov 10. PMID: 19902324.
52. Li Y, Song D, Jiang Y, Wang J, Feng R, Zhang L, Wang G, Chen Z, Wang R, Jiang Q, Liu G. CR1 rs3818361 Polymorphism Contributes to Alzheimer's Disease Susceptibility in Chinese Population. *Mol Neurobiol*. 2016 Aug;53(6):4054-4059. doi: 10.1007/s12035-015-9343-7. Epub 2015 Jul 21. PMID: 26189835.
53. Li, X., Shen, N., Zhang, S., Liu, J., Jiang, Q., Liao, M., ... & Liu, G. (2015). CD33 rs3865444 polymorphism contributes to Alzheimer's disease susceptibility in Chinese, European, and North American populations. *Molecular neurobiology*, *52*, 414-421.
54. Liu G, Zhang Y, Wang L, Xu J, Chen X, Bao Y, Hu Y, Jin S, Tian R, Bai W, Zhou W, Wang T, Han Z, Zong J, Jiang Q. Alzheimer's Disease rs11767557 Variant Regulates EPHA1 Gene Expression Specifically in Human Whole Blood. *J Alzheimers Dis*. 2018;61(3):1077-1088. doi: 10.3233/JAD-170468. PMID: 29332039.
55. Liu S, Wu Y, Liu X, Zhou J, Wang Z, He Z, Huang Z. Lack of association between MTHFR A1298C variant and Alzheimer's disease: evidence from a systematic review and cumulative meta-analysis. *Neurol Res*. 2017 May;39(5):426-434. doi: 10.1080/01616412.2017.1297340. Epub 2017 Mar 10. PMID: 28281392.
56. Liu Y, Chen Q, Liu X, Dou M, Li S, Zhou J, Liu H, Wu Y, Huang Z. Genetic Association of CHAT rs3810950 and rs2177369 Polymorphisms with the Risk of Alzheimer's Disease: A Meta-Analysis. Biomed Res Int. 2016;2016:9418163. doi: 10.1155/2016/9418163. Epub 2016 Aug 15. PMID: 27597977; PMCID: PMC5002460.
57. Liu, G., S. Zhang, Z. Cai, G. Ma, L. Zhang, Y. Jiang, R. Feng, M. Liao, Z. Chen and B. Zhao. 2013. "PICALM gene rs3851179 polymorphism contributes to Alzheimer's disease in an Asian population." *Neuromolecular medicine* 15(2): 384-388.
58. Logue, M. W., M. Schu, B. N. Vardarajan, J. Buros, R. C. Green, R. C. Go, P. Griffith, T. O. Obisesan, R. Shatz and A. Borenstein. 2011. "A comprehensive genetic association study of Alzheimer disease in African Americans." *Archives of neurology* 68(12): 1569-1579.
59. Lupton MK, Proitsi P, Lin K, Hamilton G, Daniilidou M, Tsolaki M, Powell JF. The role of ABCA1 gene sequence variants on risk of Alzheimer's disease. *J Alzheimers Dis*. 2014;38(4):897-906. doi: 10.3233/JAD-131121. PMID: 24081377.
60. Ma LL, Ou JR, Zhang W, Sun FR, Yu JT, Tan L. Single nucleotide polymorphism rs7294919 on chromosome 12q24.22 is associated with late-onset Alzheimer's disease in Han Chinese. *Neurosci Lett*. 2014 Feb 7;560:67-70. doi: 10.1016/j.neulet.2013.12.022. Epub 2013 Dec 17. PMID: 24361131.

61. Ma, X.-Y., J.-T. Yu, W. Wang, H.-F. Wang, Q.-Y. Liu, W. Zhang and L. Tan. 2013. "Association of TOMM40 polymorphisms with late-onset Alzheimer's disease in a Northern Han Chinese population." *Neuromolecular medicine* 15(2): 279-287.
62. Mao YF, Guo ZY, Pu JL, Chen YX, Zhang BR. Association of CD33 and MS4A cluster variants with Alzheimer's disease in East Asian populations. *Neurosci Lett*. 2015 Nov 16;609:235-9. doi: 10.1016/j.neulet.2015.10.007. Epub 2015 Oct 9. PMID: 26455864.
63. Medway CW, Abdul-Hay S, Mims T, Ma L, Bisceglio G, Zou F, Pankratz S, Sando SB, Aasly JO, Barcikowska M, Siuda J, Wszolek ZK, Ross OA, Carrasquillo M, Dickson DW, Graff-Radford N, Petersen RC, Ertekin-Taner N, Morgan K, Bu G, Younkin SG. ApoE variant p.V236E is associated with markedly reduced risk of Alzheimer's disease. *Mol Neurodegener*. 2014 Mar 10;9:11. doi: 10.1186/1750-1326-9-11. PMID: 24607147; PMCID: PMC3995879.
64. Miyashita, A., A. Koike, G. Jun, L.-S. Wang, S. Takahashi, E. Matsubara, T. Kawarabayashi, M. Shoji, N. Tomita and H. Arai. 2013. "SORL1 is genetically associated with late-onset Alzheimer's disease in Japanese, Koreans and Caucasians." *PloS one* 8(4): e58618.
65. Montesanto A, Crocco P, Anfossi M, Smirne N, Puccio G, Colao R, Maletta R, Passarino G, Bruni AC, Rose G. The Genetic Variability of UCP4 Affects the Individual Susceptibility to Late-Onset Alzheimer's Disease and Modifies the Disease's Risk in APOE-ε4 Carriers. *J Alzheimers Dis*. 2016;51(4):1265-74. doi: 10.3233/JAD-150993. PMID: 26923023.
66. Myers, A.J. et al. A survey of genetic human cortical gene expression. *Nat. Genet.* **39**, 1494–1499 (2007).
67. Naj, A. C., G. W. Beecham, E. R. Martin, P. J. Gallins, E. H. Powell, I. Konidari, P. L. Whitehead, G. Cai, V. Haroutunian and W. K. Scott. 2010. "Dementia revealed: novel chromosome 6 locus for late-onset Alzheimer disease provides genetic evidence for folate-pathway abnormalities." *PLoS genetics* 6(9): e1001130.
68. Nelson PT, Fardo DW, Katsumata Y. The MUC6/AP2A2 Locus and Its Relevance to Alzheimer's Disease: A Review. *J Neuropathol Exp Neurol*. 2020 Jun 1;79(6):568-584. doi: 10.1093/jnen/nlaa024. PMID: 32357373; PMCID: PMC7241941.
69. Omoumi, A., A. Fok, T. Greenwood, A. D. Sadovnick, H. H. Feldman and G.-Y. R. Hsiung. 2014. "Evaluation of late-onset Alzheimer disease genetic susceptibility risks in a Canadian population." *Neurobiology of Aging* 35(4): 936. e935-936. e912.
70. Patel A, Rees SD, Kelly MA, Bain SC, Barnett AH, Thalitaya D, Prasher VP. Association of variants within APOE, SORL1, RUNX1, BACE1 and ALDH18A1 with dementia in Alzheimer's disease in subjects with Down syndrome. *Neurosci Lett*. 2011 Jan 7;487(2):144-8. doi: 10.1016/j.neulet.2010.10.010. Epub 2010 Oct 12. PMID: 20946940.
71. Pe'er, I., Yelensky, R., Altshuler, D. & Daly, M.J. Estimation of the multiple testing burden for genomewide association studies of nearly all common variants. *Genet. Epidemiol.* **32**, 381–385 (2008).

72. Piacentini S, Polimanti R, Squitti R, Mariani S, Migliore S, Vernieri F, Rossini PM, Manfellotto D, Fuciarelli M. GSTO1*E155del polymorphism associated with increased risk for late-onset Alzheimer's disease: association hypothesis for an uncommon genetic variant. *Neurosci Lett*. 2012 Jan 11;506(2):203-7. doi: 10.1016/j.neulet.2011.11.005. Epub 2011 Nov 7. PMID: 22100662.
73. Piccio, L., Deming, Y., Del-Águila, J. L., Ghezzi, L., Holtzman, D. M., Fagan, A. M., ... & Cruchaga, C. (2016). Cerebrospinal fluid soluble TREM2 is higher in Alzheimer disease and associated with mutation status. *Acta neuropathologica*, *131*, 925-933.
74. Pontillo, A., Catamo, E., Arosio, B., Mari, D., & Crovella, S. (2012). NALP1/NLRP1 genetic variants are associated with Alzheimer disease. *Alzheimer Disease & Associated Disorders*, *26*(3), 277-281.
75. Reiman, E.M. et al. GAB2 alleles modify Alzheimer's risk in *APOE* ε4 carriers. *Neuron* **54**, 713–720 (2007).
76. Reitz, C., Cheng, R., Rogaeva, E., Lee, J. H., Tokuhiro, S., Zou, F., ... & Mayeux, R. (2011). Meta-analysis of the association between variants in SORL1 and Alzheimer disease. *Archives of neurology*, *68*(1), 99-106.
77. Rogaeva, E., Meng, Y., Lee, J. H., Gu, Y., Kawarai, T., Zou, F., ... & St George-Hyslop, P. (2007). The neuronal sortilin-related receptor SORL1 is genetically associated with Alzheimer disease. *Nature genetics*, *39*(2), 168-177.
78. Šerý O, Janoutová J, Ewerlingová L, Hálová A, Lochman J, Janout V, Khan NA, Balcar VJ. CD36 gene polymorphism is associated with Alzheimer's disease. *Biochimie*. 2017 Apr;135:46-53. doi: 10.1016/j.biochi.2017.01.009. Epub 2017 Jan 20. PMID: 28111291.
79. Sheng J, Su L, Xu Z, Chen G. Progranulin polymorphism rs5848 is associated with increased risk of Alzheimer's disease. *Gene*. 2014 Jun 1;542(2):141-5. doi: 10.1016/j.gene.2014.03.041. Epub 2014 Mar 25. PMID: 24680777.
80. Siokas V, Aslanidou P, Aloizou AM, Peristeri E, Stamati P, Liampas I, Arseniou S, Drakoulis N, Aschner M, Tsatsakis A, Mitsias PD, Bogdanos DP, Hadjigeorgiou GM, Dardiotis E. Does the CD33 rs3865444 Polymorphism Confer Susceptibility to Alzheimer's Disease? *J Mol Neurosci*. 2020 Jun;70(6):851-860. doi: 10.1007/s12031-020-01507-w. Epub 2020 Feb 22. PMID: 32088842.
81. Squitti R, Siotto M, Arciello M, Rossi L. Non-ceruloplasmin bound copper and ATP7B gene variants in Alzheimer's disease. *Metallomics*. 2016 Sep 1;8(9):863-73. doi: 10.1039/c6mt00101g. Epub 2016 Aug 8. PMID: 27499330.
82. Stocker H, Möllers T, Perna L, Brenner H. The genetic risk of Alzheimer's disease beyond APOE ε4: systematic review of Alzheimer's genetic risk scores. *Transl Psychiatry*. 2018 Aug 24;8(1):166. doi: 10.1038/s41398-018-0221-8. PMID: 30143603; PMCID: PMC6109140.
83. Takei, N., A. Miyashita, T. Tsukie, H. Arai, T. Asada, M. Imagawa, M. Shoji, S. Higuchi, K. Urakami and H. Kimura. 2009. "Genetic association study on in and around the APOE in late-onset Alzheimer disease in Japanese." *Genomics* 93(5): 441-448.

84. Toral-Rios D, Ruiz-Sánchez E, Rodríguez NLM, Maury-Rosillo M, Rosas-Carrasco Ó, Becerril-Pérez F, Mena-Barranco F, Carvajal-García R, Silva-Adaya D, Delgado-Namorado Y, Ramos-Palacios G, Sánchez-Torres C, Campos-Peña V. SORL1 Polymorphisms in Mexican Patients with Alzheimer's Disease. *Genes (Basel)*. 2022 Mar 25;13(4):587. doi: 10.3390/genes13040587. PMID: 35456392; PMCID: PMC9026506.
85. Tortora F, Rendina A, Angiolillo A, Di Costanzo A, Aniello F, Donizetti A, Febbraio F, Vitale E. CD33 rs2455069 SNP: Correlation with Alzheimer's Disease and Hypothesis of Functional Role. *Int J Mol Sci*. 2022 Mar 26;23(7):3629. doi: 10.3390/ijms23073629. PMID: 35408990; PMCID: PMC8998932.
86. van der Lee, S. J., Wolters, F. J., Ikram, M. K., Hofman, A., Ikram, M. A., Amin, N., & van Duijn, C. M. (2018). The effect of APOE and other common genetic variants on the onset of Alzheimer's disease and dementia: a community-based cohort study. *The Lancet Neurology*, *17*(5), 434-444.
87. Vélez JI, Lopera F, Sepulveda-Falla D, Patel HR, Johar AS, Chuah A, Tobón C, Rivera D, Villegas A, Cai Y, Peng K, Arkell R, Castellanos FX, Andrews SJ, Silva Lara MF, Creagh PK, Easteal S, de Leon J, Wong ML, Licinio J, Mastronardi CA, Arcos-Burgos M. APOE*E2 allele delays age of onset in PSEN1 E280A Alzheimer's disease. *Mol Psychiatry*. 2016 Jul;21(7):916-24. doi: 10.1038/mp.2015.177. Epub 2015 Dec 1. PMID: 26619808; PMCID: PMC5414071.
88. Wang KS, Liu X, Xie C, Liu Y, Xu C. Non-parametric Survival Analysis of EPG5 Gene with Age at Onset of Alzheimer's Disease. *J Mol Neurosci*. 2016 Dec;60(4):436-444. doi: 10.1007/s12031-016-0821-9. Epub 2016 Sep 1. PMID: 27586004.
89. Wang Z, Lei H, Zheng M, Li Y, Cui Y, Hao F. Meta-analysis of the Association between Alzheimer Disease and Variants in GAB2, PICALM, and SORL1. *Mol Neurobiol*. 2016 Nov;53(9):6501-6510. doi: 10.1007/s12035-015-9546-y. Epub 2015 Nov 27. PMID: 26611835.
90. Wightman, D. P., Jansen, I. E., Savage, J. E., Shadrin, A. A., Bahrami, S., Holland, D., ... & Posthuma, D. (2021). A genome-wide association study with 1,126,563 individuals identifies new risk loci for Alzheimer's disease. *Nature genetics*, *53*(9), 1276-1282.
91. Xing YY, Yu JT, Cui WZ, Zhong XL, Wu ZC, Zhang Q, Tan L. Blood clusterin levels, rs9331888 polymorphism, and the risk of Alzheimer's disease. *J Alzheimers Dis*. 2012;29(3):515-9. doi: 10.3233/JAD-2011-111844. PMID: 22258514.
92. Young, J. E., Boulanger-Weill, J., Williams, D. A., Woodruff, G., Buen, F., Revilla, A. C., ... & Goldstein, L. S. (2015). Elucidating molecular phenotypes caused by the SORL1 Alzheimer's disease genetic risk factor using human induced pluripotent stem cells. *Cell stem cell*, *16*(4), 373-385.
93. Yu JT, Mao CX, Zhang HW, Zhang Q, Wu ZC, Yu NN, Zhang N, Li Y, Tan L. Genetic association of rs11610206 SNP on chromosome 12q13 with late-onset Alzheimer's disease in a Han Chinese population. *Clin Chim Acta*. 2011 Jan 14;412(1-2):148-51. doi: 10.1016/j.cca.2010.09.024. Epub 2010 Sep 29. PMID: 20883677.

94. Yu JT, Yu Y, Zhang W, Wu ZC, Li Y, Zhang N, Tan L. Single nucleotide polymorphism rs1333049 on chromosome 9p21.3 is associated with Alzheimer's disease in Han Chinese. *Clin Chim Acta*. 2010 Sep 6;411(17-18):1204-7. doi: 10.1016/j.cca.2010.04.023. Epub 2010 Apr 26. PMID: 20427016.
95. Zhang S, Li X, Ma G, Jiang Y, Liao M, Feng R, Zhang L, Liu J, Wang G, Zhao B, Jiang Q, Li K, Liu G. CLU rs9331888 Polymorphism Contributes to Alzheimer's Disease Susceptibility in Caucasian But Not East Asian Populations. *Mol Neurobiol*. 2016 Apr;53(3):1446-1451. doi: 10.1007/s12035-015-9098-1. Epub 2015 Jan 30. PMID: 25633098.
96. Zhang X, Zhang C, Prokopenko D, Liang Y, Han W, Tanzi RE, Sisodia SS. Negative evidence for a role of APH1B T27I variant in Alzheimer's disease. *Hum Mol Genet*. 2020 Apr 15;29(6):955-966. doi: 10.1093/hmg/ddaa017. PMID: 31995180.
97. Zhang, Q. et al. Complement receptor 1 polymorphisms and risk of late-onset Alzheimer's disease. *Brain Res*. **1348**, 216–221 (2010).
98. Zhao J, Wang J, Zhao D, Wang L, Luo X. Association Between ABCA1 R219K Variant and Alzheimer's Disease: An Updated Meta-Analysis and Systematic Review. *Curr Alzheimer Res*. 2022;19(10):734-741. doi: 10.2174/1567205020666221114112838. PMID: 36380407.
99. Zhu B, Li LX, Zhang L, Yang S, Tian Y, Guo SS, Zhang W, Zhao ZG. Correlation of PICALM polymorphism rs3851179 with Alzheimer's disease among Caucasian and Chinese populations: a meta-analysis and systematic review. *Metab Brain Dis*. 2018 Dec;33(6):1849-1857. doi: 10.1007/s11011-018-0291-6. Epub 2018 Jul 23. PMID: 30039188.
100. Zhu XC, Wang HF, Jiang T, Lu H, Tan MS, Tan CC, Tan L, Tan L, Yu JT; Alzheimer's Disease Neuroimaging Initiative. Effect of CR1 Genetic Variants on Cerebrospinal Fluid and Neuroimaging Biomarkers in Healthy, Mild Cognitive Impairment and Alzheimer's Disease Cohorts. *Mol Neurobiol*. 2017 Jan;54(1):551-562. doi: 10.1007/s12035-015-9638-8. Epub 2016 Jan 7. PMID: 26742530.
